# The role of mucin-educated platelet activation in tumor invasiveness: An unfolding concern in the realm of cancer biology

**DOI:** 10.1051/bmdcn/2017070421

**Published:** 2017-11-24

**Authors:** Akbar Shoukat Ali, Arzoo Ajaz

**Affiliations:** 1 Senior Research Assistant, Maternal and Fetal Medicine, Department of Obstetrics & Gynaecology, The Aga Khan University Hospital Karachi 74800 Pakistan; 2 Pharm. D Student, Department of Pharmacy, Jinnah University for Women Karachi 74600 Pakistan

**Keywords:** Mucins, Platelets, Cancer, Metastasis

## Abstract

Metastasis is a complex and well-coordinated phenotypic transformation of cancer cells governed by aberrant genetic and molecular pathways. It has been approved as the most consistent cause of cancer death. With emerging insight into the genomics, transcriptomics and proteomics, progress has been made and reasonably large number of molecular pathways of metastasis has been forwarded, but our understanding of precise underlying molecular mechanisms remains largely scarce. It has been well-known for around a decade and more that platelets are intriguingly contributing to the cancer metastasis. However, it is only recently that cancer cells can activate platelets have started to become apparent. Surprisingly, platelets in response to cancer cell activation, supported by research observations, allow cancer cells to escape immune removal, prolong survival in vascular compartment, increased cellular adhesion and develop new cellular niches which eventually help to favor cancer metastasis. Although a widely acknowledged plausible explanation that cancer cells activate platelets to facilitate in their distant spread, the description of this remains to be confirmed. In recent years, mucins, heavily glycosylated peptide structure, have been introduced to be released by several types of cancer cells. They account for poor prognosis in wide array of malignancies, because of their significant ability to induce metastatic process. The mechanism responsible for their increased metastatic propensity remains uncharacterized, but recent work suggested the role of cancer expressed mucins in initiating platelet thrombus. The association of cancer yield mucins, platelets and metastasis therefore suggests a pressing need to explore novel molecular mechanisms and therapeutic targets thereafter.

Cancer is an umbrella term used to designate a number of diseases originating from oncogenic tissues that overcome the normal cellular mechanisms by overexpressing self-renewal and resisting growth inhibiting and death promoting signals. Cancer is recognized as a chronic, non-healing wound that strikes millions of lives around the globe.[1] Based on the WHO cancer statistics, approximately 14.1 million newly diagnosed cases and 8.2 million deaths were accounted to cancer in 2012. Further, the incidence of cancer is gauged to rise exponentially by 70% over upcoming two decades.[2] The globalization of cancer is prominently the consequence of adopting cancer-enriching, modernized lifestyle patterns such as substance abuse (smoking and alcohol use), cessation of physical activity and unhealthy dietary habits (low fruit and vegetable intake).[3] The impact of cancer extends beyond the physical and psychological realms with significant financial consequences. In addition, it enormously influences the quality of life (QoL); as a consequence the behavior of cancer patients and their family member’s decisions with regard to their treatment. [4, 5]

Metastasis entails a complex cascade of cellular and molecular events that mediate cancer cells to circulate and form secondary niche within distant tissue systems through ongoing bidirectional interaction with the host microenvironment in a manner that is conducive to survival and proliferation of cancer cells. [6] The expression of necessary characteristics such as genetic and epigenetic instability, presence of cancer stem cells, immunoevasive self-defenses, positive interaction with foreign (host) microenvironments, sustained cell cycles and chemotherapeutic antagonism sets in motion the lethal phenotypic facet of cancer; metastasis.[7, 8] Metastasis contributes to nearly 90% of cancer related deaths.[9] Patients who are diagnosed with cancers that are metastatic at presentation tend to have poor outcomes; an improved understanding of this phenomenon might provide an additional prognostic marker. Furthermore, the poor outcomes for these patients emphasize the importance of deciphering biological pathways contributing to metastasis as a means for developing novel anti-metastatic therapies. Nonetheless, the underlying molecular mechanisms governing these interactions are yet to be deciphered.

Since the discovery of Trousseau’s syndrome in 1865, platelets have been extensively trialed for their potential in advancing cancer through metastasis.[10] Platelets, besides their established role in hemostasis and thrombosis, facilitate tumor invasiveness upon activation. The reciprocity of cancer cells and platelets is critical for development of cancer metastasis. Review studies supports the published evidence that thrombocytosis is directly proportional to the increased propensity of cancer cells to express metastatic behavior and poor prognosis and life expectancy among cancer patients.[11] Studies have also documented that formation of 'platelet cloak' around cancer cells to evade immune cell cytotoxic lysis is at the mercy of activated platelets. Additionally, platelet releasates carry diverse vasogenic, mitogenic and chemotactic factors that nurture the tumor growth and support metastasis.[12] Likewise, platelets have also been implicated to gather leukocytes through chemical signals at the site of platelet-cancer cell interaction, thereby mediating smooth survival of tumor in vascular compartment and penetration to distant organs. [13]

Mucins, biochemically, are abundantly glycosylated proteins of high molecular weight with defining feature of serine and threonine amino acid tandem repeats.[14] The promising physiological functions of mucins such as cellular differentiation, cellular adhesion, cellular signaling and immune regulation make them an important part of proteomics research.[15] Conversely, their deviant expression in diseased states including inflammatory and cancerous conditions has been well reported on several occasions.[16-18] Mucins expressing human carcinomas are prone to increased metastasis and foreshadow poor clinical outcomes.[19] With the major breakthrough in molecular attributes of cancer, mucins and their association with platelet activation and cancer metastasis has assembled a lot of awareness over past few years.

Cancer cells expressing abnormal cell surface molecules, specifically the altered mucin glycoproteins are remarkable for their interaction with E, L and P-selectins. Notably, selectins also carry the potential to recognize and bind the altered carbohydrate molecules expressed by cancer cells. Certainly, such associations allow cancer cells to interact with blood borne cells including endothelial cells, leukocytes and platelets and mediate their aggressive metastatic phenotype.[20-22] Previous studies have attempted to ascertain the link between the trio of mucin, platelets and metastasis.

Platelets can be activated by mucins in a selectin-dependent manner, emphasizing the importance of mucin-selectin interaction, P and L-selectins in particular. The proposed mechanism of platelet activation by mucin is indirect and facilitated by leukocytes via interaction with L-selectin. P-selectins and their role in cancer metastasis have been the subject of extensive research over the last few years with P-selectin expression demonstrated in activated platelets.[23, 24] Furthermore, the ability of P-selectins to aggregate platelets cannot be underestimated.[25-27] A literature review suggested both *in vivo* and *in vitro* interaction of platelets with cancer derived mucins reliant on P-selectin availability. In addition, the platelet-mucin interaction without external thrombin utilization is supported by *in vivo* observation. Experimental removal of mucin from cancer cell lines significantly reduces the strength of metastasis and studies have demonstrated that tumor cell-platelet aggregation as well as establishment of tumor metastasis was attenuated in P-selectin-deficient mice.[28] This demonstrates the crucial role of hidden molecular pathways harmonized by cancer derived mucins to augment the metastatic process. Despite these exciting strides made over the last two decades, the precise mechanism of mucin platelet interaction and their role in cancer metastasis remains to be ascertained.

## 1. Conclusion

Taken together, metastasis still represents the major cause of cancer related mortality. Unfortunately, the concealed molecular pathways guiding the primary tumors to invade distant organs remain to be elucidated. Mucin, until recently, is emerging as a new accessory source for cancer to unfurl their metastatic terror. Screening of tumor with metastatic constitution is a growing concern and demands aggressive measures to attenuate its negative prognostic impact on active cases. On this account, experimental analysis will be of immense value to identify the intensity and precision of mucin contribution towards metastasis and its sensitivity and specificity both as prognostic and diagnostic molecular marker. A step towards development of accurate and reliable relationship among mucin, platelets and metastasis will eventually yield unprecedented new insights into the future therapy.

## Conflicts of Interest Statement

The authors disclose no conflicts of interest.
